# Peer researchers’ experiences of a co-produced research project on supported decision-making

**DOI:** 10.1186/s40900-022-00406-1

**Published:** 2022-12-07

**Authors:** Paul Webb, David Falls, Fionnuala Keenan, Barbara Norris, Aine Owens, Gavin Davidson, Rosalie Edge, Berni Kelly, Aisling McLaughlin, Lorna Montgomery, Christine Mulvenna, Rebecca Shea Irvine

**Affiliations:** 1 Research Department, Praxis Care, 25-31 Lisburn Road, Belfast, BT9 7AA Northern Ireland, UK; 2 Mencap NI, 5 School Road, Newtownbreda, Belfast, BT8 6BT Northern Ireland, UK; 3 School of Social Sciences, Education and Social Work, Queen’s University Belfast, 6 College Park, Belfast, BT7 1NN Northern Ireland, UK; 4 School of Applied Social and Policy Sciences, Ulster University, Magee Campus, Northland Road, Derry, BT48 7JL Northern Ireland, UK; 5 Institute for Research on Women and Gender, University of Michigan, 1136 Lane Hall, 204 S, State Street, Ann Arbor, MI 48109-129 USA

**Keywords:** Peer research, Experiential expertise, Benefits and challenges of co-production

## Abstract

**Background:**

Making decisions about your own life is a key aspect of independence, freedom, human rights and social justice. There are disabled people who, without support, would be assessed as incapable of making certain decisions but with the appropriate support are capable of making those decisions and so to not provide that support infringes their rights, undermines their autonomy and reinforces their exclusion from society. However, there is limited research evidence available about disabled people’s experiences of the range of approaches provided to support decision-making. This article will explore the experiences of four peer researchers who co-produced a research project on how people have, or have not been, supported to make their own decisions. Two of the peer researchers have experience of mental health problems and two are people with an intellectual disability. The article refers to peer research because its subject matter is the relevant lived experience of people. Peer research is therefore an approach within the broader areas of participatory research and co-production.

**Methods:**

The peer researchers interviewed 21 people with mental health problems and 20 people with an intellectual disability to gain an in-depth understanding of their experiences and preferences for how decision-making should be supported. Peer researcher experiences at each stage of the study from design to analysis were explored using data collected from the peer researchers via blogs written at early stages of the study, discussions at team meetings as the fieldwork progressed and at a final workshop at the end of the study which gave the peer researchers the opportunity to focus on their overall reflections of being a peer researcher. The article also discusses motivations to undertake the peer research role, the process of co-production and the challenges negotiated during the study.

**Results:**

The peer researchers reported a number of positive effects of being involved in the research project which included improvements in skills and self-confidence.

**Conclusion:**

The peer researchers’ involvement challenged assumptions about the inability of people with an intellectual disability and/or mental health problems to participate proactively in a research project whilst also highlighting the importance of training for all team members.

## Plain English summary

Making your own decisions is important. There are disabled people who need support to make some decisions. Supporting disabled people to make decisions is needed so that they are not excluded from society. However, the evidence is limited on disabled people’s experiences of how best to support decision-making. This article will explore the experiences of four researchers with mental health problems or an intellectual disability who worked with other researchers to conduct a project on how people have, or have not been, supported to make their own decisions. These researchers are called peer researchers. The peer researchers interviewed 21 people with mental health problems and 20 people with an intellectual disability. These interviews were conducted in order to gain a detailed understanding of the experiences and preferences for how decision-making should be supported. Blogs written by the peer researchers, discussions in team meetings and a workshop at the end of the study enabled the peer researchers to reflect on their experiences. The peer researchers reported a number of positive effects of being involved in the research project which included improvements in skills and self-confidence. The peer researchers’ involvement challenged assumptions about the inability of people with an intellectual disability and/or mental health problems to participate in a research project whilst also highlighting the importance of training for all team members.

## Background

The research was conducted in Northern Ireland (NI) where the Mental Capacity Act (NI) became statute law in May 2016, and was partially implemented in 2020. In contrast to other countries, when fully implemented, this law will replace rather than be in parallel to a mental health law. This is a unique and progressive development and a core principle of the new Act is that people are “not to be treated as unable to make a decision…unless all practicable help and support to enable the person to make a decision about the matter have been given without success” (Article 1(4)). This law is supported at an international level by the principles of the United Nations Convention on the Rights of Persons with Disabilities (UNCRPD), ratified by the UK in 2009. Under Article 12 of this Convention, State Parties must provide a range of formal and informal supports to disabled people to assist them to fully exercise their legal capacity.

Whilst these requirements are to be welcomed, there is limited evidence available about disabled people’s experiences of supported decision-making to inform the implementation of the legislation in practice. Whilst there is a growing body of literature on legal aspects of supported decision-making [7], models of supported decision-making [3] and relationships with supporters of decision-making [17], there is a more limited range of research focused on the views and experiences of people with mental health problems and an intellectual disability  [15, 15, 18]. In NI ‘intellectual disabilities’ are usually referred to as ‘learning disabilities’ but, as the audience for this article is international, intellectual disability is used. It is clear that people with mental health problems and an intellectual disability want to be involved in decisions affecting their lives and appreciate support from trusted people to reach decisions but are often restricted in exercising their own decisions by disempowering, paternalistic attitudes and practices [16, 15]. Although a range of supported decision-making approaches have been developed, as decisions become more complex, support can diminish [11]. This study was undertaken to build on the emerging body of literature on the views of people with mental health problems and an intellectual disability on supported decision-making and to contribute to an understanding of how best to implement supported decision-making under the new legislation in Northern Ireland.

Given the focus of the research on participation in decisions, it was important that the study enabled a participatory approach to involving people with mental health problems and an intellectual disability in the research process [12]. From the outset, therefore, a co-production approach was adopted involving four people with mental health problems or an intellectual disability as peer researchers.

The involvement of service users in policy and practice is now a common practice supported by local policy in NI, such as, the Health and Social Care (Reform) Act NI (2009) which provides a legal obligation for health and social care organisations to involve service users and carers in health and social care services; and globally under Articles 29 and 30 of the UNCRPD on the rights of disabled people to participate in public and political life. Alongside these policies that highlight service user involvement, there has been a strong emphasis on participatory research in both mental health and disability research over recent decades [1]. Levels of service user involvement in research have ranged from consultation or advisory roles to active participation in the design and conduct of the study [2]. Within this context, a growing number of studies relating to mental health and disability have involved people with mental health and an intellectual disability as peer researchers [9, 13]. These studies have shown how peer research can add considerable value to the research in terms of authenticity, relevance and use of personal experience. However, these studies have also identified significant challenges related to resourcing, recruiting and supporting peer researchers [2, 9]. It is within this context that the peer research methodology reported here was developed.

Whilst other literature on peer research has sought to examine the benefits and challenges of peer research, this article will provide a more detailed focus on the actual experience of the peer researchers as they navigated their involvement in the study, from the early stages of recruitment and training to the latter stages of dissemination and impact. This exploration of the journey of the peer researchers as the research process unfolded will highlight what helped to make it a positive experience but also how to enhance approaches to peer research with people with mental health and an intellectual disability in future studies. Throughout the article draws on data collected from the peer researchers based on blogs written at early stages of the study, discussions at team meetings as the fieldwork progressed and a final workshop at the end of the study focused on their overall reflections on being a peer researcher. The article also conceptualises peer research as an approach within the broader areas of participatory research and co-production so that all team members felt they ‘belonged’, were integral to, and were able to make a contribution to the project’s success [10, 18].

## Methods

The aim of the peer research approach was to ensure that the study design, method, data collection and analysis was informed by the expertise of those with lived experience of mental health problems or an intellectual disability [4]. It was expected that this level of co-production would improve the accessibility of the interview approach, encourage participant engagement, provide deeper insight from a service user perspective, and add to the impact of dissemination. The team were also hopeful that it would have some benefits for the peer researchers in terms of training, experience and empowerment [14]. Collaboration between team members was fostered through monthly team meetings and by making decisions consensually. Joint data collection involving peer researchers and academic researchers was the norm.

Four peer researchers were recruited through an open recruitment process led by Praxis Care and Mencap (partnering voluntary sector organisations who support people with mental health problems and an intellectual disability). The peer researchers were recruited using standard recruitment practices by the voluntary sector partners. An agreement with the peer researchers was reached whereby only broad information about everyone’s background would be included. A two-day peer researcher training programme was delivered by academic researchers on the team from Queen’s University. Training focused on building the peer researchers’ capacity and confidence covering: roles and responsibilities; research methods; interviewing skills; research ethics; self-care; analysis; and report writing. The training also included role-plays and reflection to help prepare for a range of potential scenarios during fieldwork. Semi-structured interviews were used to find out about people’s experiences of supported decision-making and peer researchers collaborated with the wider team to devise appropriate interview questions, including an adapted accessible version of the interview schedule.

Praxis Care and Mencap led on the purposive selection of forty-one participants ensuring a range of experiences and a broad demographic profile (e.g. gender, age). Participants were invited onto the study by phone/email/talking directly with a staff member familiar with the study about the level of involvement expected. With consent, participants took part in an interview with a peer researcher. Peer researchers and participants were matched based on experience of mental health problems or intellectual disability. The lead in each organisation played an important role in this process and also ensured that peer researchers were informed about the communication needs and preferences of participants prior to interviews. The interview was structured around three key areas: experiences of making decisions, approaches to support and ideas for future support. Prompt sheets were used as visual aids to support the interview process, visually presenting questions and possible responses in a clear, concise and accessible format. Peer researchers were accompanied to interviews by another researcher on the team who provided transport and support, if needed. Prior to starting the interviews, the accompanying researcher read through the ‘participant information sheet’ with the participant reminding them of the purpose of the study and what was expected before written consent was given. Easy read versions of the information sheet and consent form were developed in partnership with the peer researchers. The peer researcher then led the interview with support, if necessary, from the accompanying researcher. All participants provided consent for the interviews to be audio recorded. These were transcribed and anonymised for analysis. An initial coding frame was developed by a peer researcher and an academic researcher based on a thematic analysis of a sample of transcriptions, independently identifying codes and then discussing them, using the qualitative data software NVivo. This coding framework was then shared with the other peer researchers and the wider research team who made amendments/additions.

Analysis also took place at a final workshop with the focus of analysis being informed by the peer researchers’ blogs and regular team meetings. Three of the peer researchers continued to work with the wider research team to identify the sections described in the article, recommendations from the findings, develop easy read versions of the final report and to disseminate the findings to a range of audiences including presentations at a launch event and a participant workshop where they presented the findings to all those who were interviewed. The peer researchers were also ‘key note’ speakers at a conference within N. Ireland. The responses to the peer researchers’ participation in the conference were very positive.

## Results

### Beginnings: recruitment, training and preparing

The motivating drivers for the peer researchers to undertake the role were twofold: to use their personal and professional experience to enable people with mental health problems and an intellectual disability to have a voice; and to develop their own experience and skills:

Wanting to help people... be more open, to help them understand that there’s help out there too… The thing that motivated me is probably speaking up for people with learning disability in my local area… I haven’t really spoke much but I have gotten better at speaking about things now.

To make sure other people had that opportunity to be supported, to do what’s best for them… because I’d been in a position where I was supported to make a decision when the decision could very easily have been taken out of my hands.

For one peer researcher the project offered her the first experience of paid employment and for another it provided a route back into paid employment:

The other motivation was to get back into… employment that was in a very safe and controlled environment, you know rather than being dropped into a big corporation like I was before so really to build up your confidence to get me back to employment.

Peer researchers were particularly encouraged to apply for the position when they saw that the essential criteria for the post included experience of mental ill health or learning disability:

On the job advertisement, it said that you must have a current or past mental ill health and I was like, YES! I thought they’re actually going to see it as an asset instead of a disadvantage for the job. So that was like “Oh that sounds like the type of people I want to work for”.

Another peer researcher also explained that the level of support provided by the research team was also an important factor in her decision to apply for the role:

I knew I’d be getting support where a regular job could be a challenge.

From the outset of the project, the peer researchers had a range of hopes and fears related to the role. They hoped to develop their research skills and confidence but also to have a positive impact on the accessible design of the study and its overall impact:

One of my expectations would be to… help those ones who we were interviewing because… the ones who we interview might not be able to understand you.

I would expect to get some sort of hope out of doing it. From interacting with more people… being taken out of my comfort zone quite a bit, so I went in with the expectation, the hope really that there’ll be positive outcomes.

Peer researchers were also anxious about what their role would be but these anxieties were diminished by the training programme. One peer researcher wrote the following blog about her experience of the two-day peer researcher training programme:

I thought the training was very interesting and very well explained... We now have a better understanding of the role we will be taking up... and the roles the rest of the team... I really did enjoy it and because it was interactive... and everyone had an input. I felt really relaxed during the training and everyone in the team is nice and friendly... I learnt more about the project and I now know what some of the big words mean. I really feel positive about the role now training is finished.

The peer researchers also explained that the inclusive approach to training was important as it showed respect for the contribution of the peer researchers and allayed fears of tokenism:

Before the training I’d been a bit unsure about what the level of involvement was going to be, whether it was going to be a just token thing, you know to tick the box to get funding… which thankfully it hasn’t been. It’s been the complete opposite of it… The training wasn’t kind of something that was forced on us. It was a dialogue and you could see that the rest of the team were listening to what I said so it was quite a nice. It really was a team… so it was really good.

The training was also a learning opportunity for the academic team members who were challenged to deliver the content in a way that was accessible for everyone. One of the peer researchers explained how they helped the academic researchers to adapt the pace of their approach to make it more accessible:

It was good to get to know the people from Queen’s… the last time we saw the people from Queen’s, they went backwards and forwards, backwards and forwards, very very fast and one of us was just saying “Will you calm down and explain to < name removed > and < name removed > sort of thing?”… So… we could relate to their topic.

### Co-production: designing and conducting interviews

With regard to the semi-structured interview schedules which the peer researchers helped to design, one peer researcher was concerned about the wording of a question and helped the team to re-phrase the question in a more sensitive manner. The peer researcher therefore identified an issue which the academic researchers had not identified:

There was one of the questions at the training I kind of vetoed and was like ‘I’m not asking that’ because that could traumatise people a wee bit and it could then impact me as well… I thought “Oh dear if you ask some people that, with mental health - that could just be a can of worms”… So the way the question was put was very, very different then. But it was nice to have that level of involvement and be able to air my concerns and be listened to.

Similarly, another peer researcher described developing shorter, more accessible questions for participants with an intellectual disability and allowing more time for these interviews:

We did come up with questions people would be willing to answer but not to bombard them with too many as well and not to make them too long so they don’t get bored.

Prompt sheets were also used during interviews where needed. The prompt sheets included the visual presentation of questions together with clear response options. This level of accessibility was important for participants but also for the peer researchers, especially those with an intellectual disability:

Reading and listening [was important] in interviews because some people ask you to repeat some questions so you couldn’t really go too far down the page. So it was a good because the pages were easy read, you didn’t really get lost in them… They were just the right length for some people.

Throughout the interviews, peer researchers found they had to adapt their approach to the individual circumstances of the individual and their environment and, in some cases, this was challenging. Peer researchers recalled feeling initially unsure how to address unexpected disruptions to interviews, for example, coping with background noise. In some cases, they found it frustrating when participants had forgotten about the interview and it had to be re-scheduled or when participants focused on issues not relevant to the core interview questions, as two peer researchers explained:

Some interviews were a bit challenging. I remember one person who… was very talkative about his mum in any answer... It was a bit challenging… but I still managed ok… There was one where the person was just looking to show photos… but they still answered those questions really well.

It was kind of like they were trying to avoid a topic that you were trying to talk about… On a couple of our interviews when we got to the place the people forgot… change could happen on the day. I used to be bad with changes but I’m getting there.

It was also challenging to hear personal stories, however, peer researchers emphasised the importance of listening to these narratives:

People kept telling you their personal life. Sometimes it could be very difficult for them but you have to show them that you’re listening.

In most cases, peer researchers had some prior information about the person’s communication preferences, however, there were situations where peer researchers felt they would have benefitted from further advance information about the needs and preferences of the participant:

I must admit that’s what I found tough. Obviously people are entitled to their privacy but having a slight indication of what you were maybe going to be facing would have been helpful [to] know how to set the tone of interviews… If someone… was able to say like something like “Don’t maintain eye contact”… wee things that might have helped a bit… or even if the staff could say “Oh so and so is having a good day or a bad day today”.

Given these challenges, debriefing with the accompanying researcher was helpful although not always necessary:

She [accompanying researcher] always made sure at the end of the interview, was everything ok and I was always ok. There wasn’t any harming stories but there were some difficult stories.

The peer researchers developed confidence in their skills and ability to respond to fieldwork challenges as they gained more experience of interviewing:

I think there was some challenges along the way but you know working through them has led more positives. They were just temporary stumbling blocks but I’ve learnt then, as the interviews were going along, I learnt to take in the knowledge from the previous interviews and adapt a wee bit.

The peer researchers considered their personal experiences of mental health problems/an intellectual disability to be a major benefit in the interview process. They found that, when they explained to participants that they were employed as peer researchers who had personal experience of mental health problems or disability, they established a connection with participants that enabled an open channel of communication and instilled a sense of hope for some participants:

Having the experience of being supported to make a decision, I felt gave me a pretty good understanding of what we were setting out to do… At some of the interviews, as soon as I mentioned my background, that I wasn’t an academic… I was a peer with mental health issues, with two people in particular, that just broke down so many barriers because they kind of opened up more because I wasn’t an authority figure… and they kind of could understand that I could see where they were coming from more. So I think that helped in those cases… They knew that someone like me could relate to them maybe that bit more… They were almost more interested in me and how I’d got to the stage where I was at… The last person I interviewed in particular was like “Oh that gives me some hope now that in a year’s time maybe I can be you know, further on”.

This personal experience was augmented by the peer researchers’ additional experience of having a social network comprising other individuals with mental health problems or intellectual disability which widened their knowledge of the salient issues for this service user group and the skills required to engage them in the research process:

Most of my friends in my local area and the ones I’ve done research for which involve the community and mostly people with learning disabilities so that helped too.

Critics of peer research have previously highlighted concerns that peer researchers may not have the skills to deal with ethical dilemmas that arise during fieldwork, including participant distress (Bigby, [2]). In contrast, the peer researchers in this study were acutely aware of the risk of emotional harm to the participant and themselves. Peer researchers suggested that their own personal experiences equipped them with key skills for identifying distress and intervening early to offer a break or access to support, if necessary. This was apparent in the re-designing of questions so they were worded more sensitively but also in the ways in which peer researchers recognised and responded to potential signs of uneasiness during interviews:

It was knowing when not to push it because you could sense people getting agitated at times… I just knew some tell-tale signs that I would have had, that they had, so I think having that knowledge of mental health helped to know when not to… Maybe I was more cautious going into it…. that there could be anxiety there of talking to someone new and people could get a bit worked up about things so I took quite a softly approach.

Given the fieldwork challenges, regular team meetings were held to report on study progress. Staff from the partnering organisations also met with the peer researchers to debrief/offer support and to ensure consistent communication and dialogue with peer researchers and address self-care needs:

The continuing meetings were good… so we’ve been kept in the loop so throughout it all… It’s about the support that’s been in place as well. You wouldn’t get a lot of places from the very start just even talking about self-care plans… Our experience has been seen as assets but there has be recognition that you may still need support so that’s been quite good.

### Endings: data analysis and ongoing co-production

Analysis involved the development of a coding frame and subsequent thematic analysis within NVivo. The coding frame was developed by a peer researcher and an academic researcher using a sub-set of transcripts. Amendments and/or additions to the coding frame were then made by the other members of the research team including peer researchers. Involving each peer researcher in different ways at this stage of analysis demonstrated an ongoing commitment to facilitating co-production and sharing ownership of the project at later stages of the research process:

We met and read through what you already analysed... It was in themes and if there was ever anything missing we told the coordinator… We thought that would have been the best way of doing it.

Some previous peer research studies have only involved peer researchers in the data collection process with data analysis being undertaken by academics with analytical expertise. In this study, the involvement of peer researchers in the data analysis was critical as they could identify themes in the transcripts that the academic researcher may not have observed. One peer researcher, who co-worked on the initial data analysis with an academic, commented on this process and the use of multiple peer research experience to inform data analysis:

For the coding framework we might have seen themes in places where other people wouldn’t have… knowing some of the lingo… might help identify some themes. And I think having so many different perspectives involved… helped quite a bit as well because obviously if I was looking at transcripts I would only really understand probably more the mental health ones and the learning disability transcripts I wouldn’t pick up a lot of things in so I think it has been helpful having people with these backgrounds involved too.

This level of involvement in analysis was greatly appreciated by peer researchers who had previously been involved in research but not given full credit for the outputs from the study:

My dealings with academics… previously was that they would never let anyone take credit for anything whereas the credit is actively being directed to us so that was completely mind blowing… It was good because you felt really valued then, you know that you’re trusted to be able to, you know, talk on their behalf… I just wasn’t used to a lot of academics sharing credit… I’m still sort of pleasantly surprised at how inclusive it was and the level of involvement we had… We’ve been incredibly involved with it and pretty much every decision that’s been made we’ve had input… It has made you more committed to the project and more interested and you’ve bought into it more so you want to see the outcomes and see it through… It’s been very empowering by being that involved and getting the responsibility and trusted with the responsibility to do the job.

It is very inclusive and empowering to our needs but also to each other, which you wouldn’t have in many other jobs.

Three peer researchers also maintained their involvement with the study through to project completion and were therefore heavily involved with the development of recommendations, an easy read final report and with dissemination. Involvement at these later stages of the study were important to the peer researchers as it gave them an opportunity to have a voice at stakeholder events and to deliver impactful messages from the study to policy makers and service providers.

At the end of the study, the peer researchers participated in a workshop facilitated by one of the academic researchers to focus on reflections on their overall experience and next steps. The peer researchers were very positive about the interpersonal and presentational skills they had developed during the study. Indeed, based on their experience, two peer researchers had been offered positions on advisory groups and another had secured a research post:

It was good and interesting. I learned new skills of different ways of talking and approaching people… There’s another opportunity that I’ve been given I probably wouldn’t have got if I didn’t do this. It’s a service user forum for another area.

Other longer term benefits from being involved in the study included the establishment of new social and community networks, being empowered by the sense of achievement of successfully completing the peer research project, and feeling a sense of contribution to a project that may have a valuable impact on people’s lives. Peer researchers also noted that the project had given them a new perspective on their own personal experience of mental ill health and disability and encouraged them to see their experiences as an asset of value to a range of projects:

It was a new perspective of self… Before the project started I guess I was just someone with mental health issues and that was a disadvantage whereas this involvement has been an asset because I’ve been able to use it in a positive way. So being able to kind of just get that more positive outlook on myself has been quite a big benefit from it.

## Discussion

The breadth and quality of data collected is evidence of the success of the peer research approach which facilitated the collection of rich data that provides insight into the experience of supported decision-making.

In alignment with the principles of supported decision-making, the experience of the peer researchers challenges assumptions about people with disabilities and demonstrates their capacity to fully participate at each stage of the research process, with appropriate training and support. However, the peer research experience also shows that genuine participation does not always mean everyone is participating in the same way and at the same level at every stage. At times, peer researchers were leading, at other times advising, contributing ideas and critiquing themes. Indeed, allowing for changing roles and different ways to participate empowered the peer researchers and ensured their inclusion at all stages of the research process in a way that was responsive to their preferences and skill set [8].

Roles in the research process differed depending on the preferences and strengths of the individual peer researcher and factors such as their mental health/intellectual disability and the number of hours they were permitted to work (without affecting disability benefit/other employment). For example, the team decided that one peer researcher with experience of mental health problems would work in partnership with an academic researcher on the initial analysis of data as he had prior experience of analysing research data and the two peer researchers with an intellectual disability did not feel it played to their strengths to read transcripts and analyse dense text. Instead, they preferred to comment on the initial code book and suggest additions or changes to emerging themes. In this way, they provided an internal validation of the identified themes based on their own personal experiences and experiences of the interviews that they had carried out. The fourth peer researcher also contributed her views on the code book but ended her participation in the study at this point before dissemination activities began due to other personal commitments. The flexibility to respond to individual needs and circumstances is important in peer research and reflects the more unpredictable nature of co-production with each team member contributing at a pace and level that is appropriate at various stages of the project.

Utilising the expertise of all team members was a key feature of the study that allowed each member to feel they were making a valued contribution to the process. Service users brought personal experience, previous related skills and a growing confidence in research skills. Partnering organisations contributed much to the recruitment and coordination of the study, whilst also supporting individual peer researchers and participants. Academics contributed their research expertise in terms of study design, training and supporting peer researchers and assisting with analysis and write-up of findings. Engagement in the peer research process was also beneficial to the academics and partnering organisations involved in terms of developing greater insight into service user perspectives and shared learning about co-production. The inter-dependence of the research team, drawing on a combined skill set, greatly strengthened the study.

At the peer research workshop, the team reflected on the recommendations they would have for other researchers considering a peer research approach to mental health or disability studies. The peer researchers emphasised that they would strongly encourage other people with mental health problems and an intellectual disability to consider a peer research role and would like to raise awareness of the benefits of peer research and the process involved:

It’s getting the word out to people about what peer research involves and the benefits of it could maybe encourage more people to get involved in peer research because I think from a mental health point of view it’s kind of quite therapeutic being involved in it.

I would say for future researchers don’t let your mental health or learning disability put you off... If you believe in it, you can do it.

However, peer researchers also highlighted that future projects must provide robust training and support for peer researchers to prepare them for the challenges of fieldwork:

The training made us more aware what we would dealing with so I would say if someone was going for a similar project they would need to do some training to be prepared more as there are some interviews that can be overwhelming and challenging.

Indeed, academics also require training on co-production, accessibility and how best to support peer researchers with mental health problems or an intellectual disability. Working in partnership with peer researchers requires a commitment to sharing power and a flexible approach to the design and development of the research process. Holding regular team meeting and cultivating positive professional relationships during every interaction also meant that the team members were able to assess what levels of support might be required. These values and skills are relevant to both peer researchers and academics involved in co-produced research.

Peer researchers particularly emphasised the importance of being involved at all stages of the research from the design of research questions to fieldwork, data analysis and dissemination. This level of involvement helps to avoid tokenistic approaches to co-production and ensures that peer researchers have full opportunity to influence the research and contribute fully to the study. For this study, the peer researchers were recruited after the funding for the study had been secured. It would be ideal if peer researchers could also be involved in the initial application process, and there are now an increasing number of people with experience as peer researchers who would be able to do this, but how that would be funded remains a challenge.

Our study benefitted greatly from the involvement of voluntary organisations who assisted with participant recruitment and matching, the coordination of fieldwork and support for peer researchers. Future peer research studies would benefit from firm partnerships with agencies who have strong networks in the community and who have experience of supporting people with mental health problems or an intellectual disability. Our experience of co-production was grounded in a mutual commitment to researching the lived experience of people with mental health problems or intellectual disability to develop recommendations for improving approaches to supported decision-making. The benefits for everyone involved were clearly evidenced, and the opportunity for peer researchers to make a difference was concisely captured by a peer researcher who said:

It was being involved in something and feeling useful and that you’re actually contributing something has been quite good because you know the outcome of the project is going to have real impact on people’s lives.

## Conclusion

The experience of the peer researchers challenged traditional assumptions about the inability of people with an intellectual disability and/or mental health problems to participate proactively as part of a research team. The peer researchers reported a number of benefits to peer research which included improvements in skills and confidence as well as a sense of empowerment. They also reported appreciating how their experiences as people with mental health problems and/or an intellectual disability were valued. The peer researchers’ experience with mental health problems and/or an intellectual disability also helped establish a rapport with participants which meant that they could elicit high quality responses during each interview. Flexible roles within the team meant that the preferences and skills sets of peer researchers were fully utilised. Training for all team members was essential as were regular meetings and de-briefing with peer researchers after each interview. Effective, multi-agency partnership working which included peer researchers, academics and staff working in services was seen as essential to the success of this co-produced research project. Retaining peer researchers in supported employment on completion of research projects remained an issue which meant that it was difficult to involve peer researchers in the initial application process. This gap in funding needs to be addressed in order to ensure that research projects are maximally co-productive from development of the application through to knowledge mobilisation and beyond.

## Data Availability

The full dataset is not available as, even when anonymised, there is the potential for people to be identifiable given the relatively small number of people and services involved.
